# Diagnosis and treatment of carotid body paraganglioma: 21 years of experience at a clinical center of Serbia

**DOI:** 10.1186/1477-7819-3-10

**Published:** 2005-02-12

**Authors:** Lazar B Davidovic, Vojko B Djukic, Dragan M Vasic, Radomir P Sindjelic, Stevo N Duvnjak

**Affiliations:** 1 Institute for Cardiovascular Diseases, Clinical Center of Serbia, Belgrade, Serbia and Montenegro; 2 Institute for Otorhinolaringology and Maxillofacial Surgery, Clinical Center of Serbia, Belgrade, Serbia and Montenegro

## Abstract

**Background:**

The carotid body paraganglioma (chemodectoma) is a relatively rare neoplasm of obscure origin. These are usually benign and commonly present as asymptomatic cervical mass.

**Patients and methods:**

Records of 12 patients (9 female and 3 male) with carotid body tumors treated between 1982 and 2003, treated at our center were retrospectively reviewed. Data on classification, clinical presentation, and surgical treatment were extracted from the case records. Surgical complications and treatment outcome were noted and survival was calculated by actuarial method. The literature on carotid body paraganglioma was reviewed.

**Results:**

The average age of the patients was 52 years (range 30–78 years). Eight of these cases presented as a large asymptomatic non-tender neck mass, and two each presented with dysphagia, and hoarseness of voice. As per Shamblin classification seven of tumors were type II and 5 were types III. In 7 cases subadventitial tumor excision was performed, while in 5 associated resection of both external and internal carotid arteries was carried out. The artery was repaired by end-to-end anastomosis in one case, with Dacron graft in one case, and with saphenous vein graft in 3 cases. There was no operative mortality. After a mean follow-up of 6.2 years (range 6 months to 20 years), there were no signs of tumor recurrence in any of the cases.

**Conclusions:**

Surgical excision is the treatment of choice for carotid body paragangliomas although radiation therapy is an option for patients who are not ideal candidates for surgery. For the tumors that are in intimate contact with carotid arteries, the treatment by vascular surgeon is recommended.

## Background

Paraganglioma arising from the carotid body are relatively rare tumors but constitute majority of head and neck paragangliomas (60–70%) [1-6]. The term paraganglia was first used by Kohn in the early twentieth century and is the most appropriate nomenclature from an embryologic standpoint [3-5]. Other terms such as carotid body tumor, glomus tumor, chemodectomas, and nonchromaffin tumor are less accurate terms and therefore should be best avoided [7-13]. The neoplasm present as asymptomatic neck mass. We report our experience with surgically treated carotid body tumors.

## Patients and methods

Between 1982 and 2003, 12 patients (9 female and 3 male) with carotid body paraganglioma were surgically treated at the Institute for Cardiovascular Diseases, Serbian Clinical Centre. Mean age of the patients was 52 years (range 30–78 years). The records of these patients were retrospectively reviewed for clinical presentation, diagnostic work-up, surgical treatment, and outcome. Descriptive data was presented as frequency and percentage. Survival was calculated by actuarial method. World literature on carotid body paraganglioma was reviewed. The articles were extracted using the key words carotid body and paraganglioma.

All the patients were followed-up periodically every 6 months for the first year, yearly for next 5 years, thereafter only select patients were followed. The patients who had undergone carotid artery repair were followed-up with yearly duplex scanning; two patients were followed by a computerized tomography (CT) scan, and one by regular magnetic resonance (MR) imaging.

## Results

**Figure 1:**
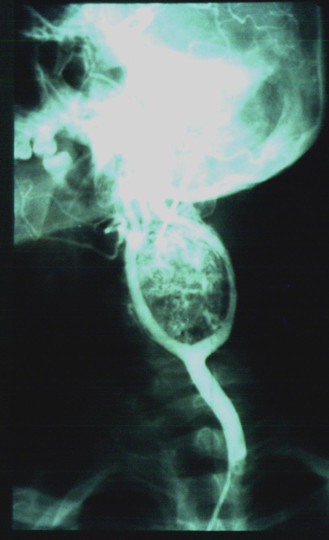
Selective carotid angiography showed carotid body paraganglioma. The typical separation (''lyre sign") of external and internal carotid arteries, are presented.

**Figure 2:**
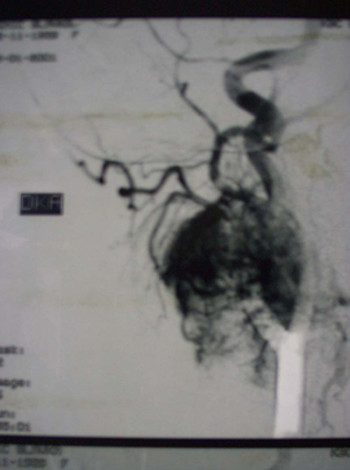
Selective carotid angiography showed hypervascularization of the carotid body paraganglioma mostly from the external carotid artery.

Eight cases presented as large non-tender neck masses located just anterior to the sternocleidomastoid muscle, two patients presented with dysphagia due to hypoglossal nerve compression, while two other had hoarseness of voice. The duplex ultrasonography and selective carotid angiography were used for diagnosis in the eleven cases, CT in five, and MR imaging in three cases (Figure 1 and 2). In one case the diagnosis was established on intraoperative exploration.

Intraoperatively on exploration of the neck seven of the cases showed a medium size tumor intimately associated and compressing carotid vessels (Shamblin II), and a large tumor involving carotid vessels in five cases (Shamblin III). In 7 cases of Shamblin II carotid body paraganglioma a subadventitial tumor excision was performed while in other 5 cases both external and internal carotid arteries were resected. One of these was repaired by end-to-end anastomosis, one with interposition of Dacron^® ^graft, and other 3 were repaired with reversed saphenous vein graft.

The histological examination showed no signs of malignancy in any of the tumors. In two cases transient hypoglossal nerve palsy was noticed. Another patient had unexpected postoperative hoarseness of voice due to the transient vagus nerve palsy. All these three cases subsequently recovered. There was no operative mortality. The patients were followed-up from the 6 months to 20 years (mean 6.2 year) no local, regional or distant metastasis was noticed. The actuarial survival was 100%.

## Discussion

The carotid body was first described by von Haller in 1743 [14]. It is highly specialized organ located at the common carotid artery bifurcation. Its feeding vessels run primarily from the external carotid artery. The function of the carotid body is related to autonomic control of the respiratory and cardiovascular systems, as well as blood temperature [3,10,12,15-23]. Paraganglioma is a relatively rare neoplasm occurring in carotid body [1-6].

The carotid body paraganglioma is more common in women [2-5,20,25-28]. The incidence of bilateral carotid body lesions is approximately 10%. Most of these lesions are benign however malignant behavior is often encountered. For diagnosis of malignant carotid body paraganlioma there are no clear histological characteristics that differentiate it from benign lesions. This diagnosis is reserved for the tumors with local, regional and distant metastasis. The rate of malignancy is reported to be 6–12.5% of all cases [3-5,9,11,29-35]. The 7–9% of the cases are hereditary [2,4,20,25-28,36]. None of our cases were bilateral or hereditary.

Carotid body paraganglioma often present as slow growing, non-tender neck masses located just anterior to the sternocleidomastoid muscle at the level of the hyoid. The tumor is mobile in the lateral plane but its mobility is limited in the cephalocaudal direction [3-5,13-15,21,26,36-38]. Occasionally the tumor mass may transmit the carotid pulse or demonstrate a bruit or thrill [39]. Because of its location in close approximation to carotid vessels and X-XII cranial nerves, tumors enlargement causes progressive symptoms such as dysphagia (two of our cases), odynophagia, hoarseness of voice (two of our cases) or other cranial nerve deficits [2-5,14,26,27,32,37,40]. The patients may give a history suggestive of symptoms associated with catecholamine production such as fluctuating hypertension, blushing, obstructive sleep apnea and palpitations [3-5,10,14,15,21-23,37].

Size of the tumor has a great importance not only for its clinical manifestations but also for treatment. In 1971, Shamblin introduced a classification system based on tumors size [41]. They classified small tumors that could be easily dissected away from the vessels as group I. Group II (7 of our cases) included paragangliomas of medium size that were intimately associated and compressed carotid vessels, but could be separated with careful subadventitial dissection. Group III consisted of (5 of our cases) tumors that were large and typically encased the carotid artery requiring partial or complete vessel resection and replacement. Histologically, carotid body paraganglioma resemble the normal architecture of the carotid body. The tumors are highly vascular, and between the many capillaries are clusters of cells called Zellballen [41].

The carotid angiography is the most useful diagnostic test for paragangliomas. The angiography demonstrates tumor blood supply and widening of the carotid bifurcation by a well-defined tumor blush ("lyre sign"), which is classic pathognomonic angiographic finding [5,8,37-39,42,43]. MR and contrast CT are more effective non-invasive imagining modalities comparing with duplex ultrasonography, especially for small tumors [3,37-39,42-45]. Radioimmunodetection of carotid body paraganglioma by ^111^In labeled anti-CEA antibody is also described in literature [9,46]. The differential diagnosis includes other tumors in this area, carotid artery aneurysms and elongation. For this reason using of precutaneous fine-needle aspiration for preoperative diagnosis of carotid body paraganglioma, can be very dangerous [47].

Resection of carotid body paraganglioma carries inherent risks of injury to the cranial nerves, carotid arteries as well excessive blood loss. Reigner first attempted resection of a carotid body paraganglioma in 1880, but the patients did not survive [48]. Maydel was the first to remove a carotid body paraganglioma successfully in 1886, but the patient became aphasic and hemiplegics due to internal carotid artery ligature [49]. In 1903, Scudder performed the first successful removal of carotid body paraganglioma [50]. The surgical excision with careful subadventitial dissection is treatment of choice for most carotid body paragangliomas (Shamblin I and II) [2-6,14-18,34,37-40,43]. The Shamblin III of carotid body paraganglioma requires resection of the external and/or internal carotid artery. If the internal carotid is encased in tumor or damaged during resection, immediate repair/replacement should be performed [15,37,39,40,42,43,51,52]. The second problem during tumor excision is bleeding, which sometimes can be massive. In such cases clamping of all carotid arteries is useful, with placement of internal carotid shunt [18,35,37]. Having in mind our experience with surgical treatment of both carotid body gangliomas as well as carotid stenosis, we recommend Pruitt-Inahara double balloon occlusive internal carotid shunt [37]. The placement of this shunt through incision on the common carotid artery contributes to the adequate bleeding control from the common and internal carotid arteries, as well as brain protection. This procedure gave a clean and dry operative field during tumor removal [3,37]. Some other articles recommend angiographic embolization preoperatively [3,23,37,42,53-55]. The Preoperative embolization of a carotid body paraganglioma can be performed by ethanol or polyvinyl alcohol. The finally result is a complete devascularization [55]. Earlier the carotid body paragangliomas were considered radioresistant [34]. However, more recent studies indicate good responses to radiation therapy [11,30]. Most authors recommend radiotherapy for giant and recurrent carotid body paragangliomas, and with malignant carotid body paragangliomas metastatic to the regional lymph nodes [8,33-36].

The modern surgical techniques have reduced the risk of postoperative stroke in carotid body paraganglioma resection to less than 5% [37,40,56]. However, the incidence of cranial nerve injury remains strikingly high, ranging from 20% to 40% [37,38,48,56,57]. In 20% of patients the neurological deficits is permanent. We found two (18%) transient hypoglossal, and one transient vagus nerve damage. recurrence after complete resection occurs in approximately 6% of patients [15,37,39,40,42,43,51,52]. In our study however, there were no recurrences. The patients with internal carotid artery reconstruction should undergo duplex scanning periodically to identify graft stenosis.

## Conclusion

Early operative management is warranted to avoid the possibility of eventual metastasis and progressive local invasion to the point of inoperability. In case of tumors intimately contact with carotid arteries, the treatment by vascular surgeon is recommended.

## Competing interests

The author(s) declare that they have no competing interests.

## Funding source

Nil

## Authors' contributions

**LBD: **Preperation of draft manuscript

**VBD: **Literature search, data collection

**DMV: **Study design, data analysis, interpretation, preparation of draft

**RPS: **Study coordination, data interpretation, manuscript preperation

**SND: **Manuscript editing, preparation of final manuscript for publication

All authors read and approved the manuscript.
